# Experience of the Coronavirus Disease (COVID-19) Patient Care in the Amsterdam Region: Optimization of Acute Care Organization

**DOI:** 10.1017/dmp.2020.446

**Published:** 2020-11-19

**Authors:** Eva Berkeveld, Sarah Mikdad, Harmen R. Zandbergen, Adriaan Kraal, Maartje Terra, Mark H. H. Kramer, Frank W. Bloemers

**Affiliations:** 1 Department of Trauma Surgery, Amsterdam University Medical Centers, Location VUmc, Amsterdam, The Netherlands; 2 Department of Cardiothoracic Surgery, Amsterdam University Medical Centers, Location VUmc, Amsterdam, The Netherlands; 3 Landelijk Coördinatiecentrum Patiënten Spreiding (LCPS), IG&H Consultancy, Utrecht, The Netherlands; 4 Department of Internal Medicine, Amsterdam University Medical Centers, Location VUmc, Amsterdam, The Netherlands

**Keywords:** coronavirus disease, COVID-19, hospital admission capacity, pandemic

## Abstract

The coronavirus disease (COVID-19) pandemic causes a large number of patients to simultaneously be in need of specialized care. In the Netherlands, hospitals scaled up their intensive care unit and clinical admission capacity at an early stage of the pandemic. The importance of coordinating resources during a pandemic has already been emphasized in the literature. Therefore, in order to prevent hospitals from being overwhelmed by COVID-19 admissions, national and regional task forces were established for the purpose of coordinating patient transfers. This review describes the experience of Regionaal Overleg Acute Zorg (ROAZ) region Noord-Holland Flevoland, in coordinating patient transfers in the Amsterdam region. In total, 130 patient transfers were coordinated by our region, of which 73% patients were transferred to a hospital within the region. Over a 2-month period, similarities regarding days with increased patient transfers were seen between our region and the national task force. In parallel, an increased incidence in hospital admissions in the Netherlands was observed. During a pandemic, an early upscale (an increase in surge spaces) of hospital admission capacity is imperative. Furthermore, it is preferred to establish national and regional task forces, coordinated by physicians experienced and trained in handling crisis situations, adhering full transparency regarding hospital admission capacity.

The coronavirus disease (COVID-19) pandemic has spread rapidly across the globe, already claiming lives of over a quarter-million people at the moment of writing.^1^ Currently, the surging number of COVID-19 cases in need of acute specialized care pushes hospital capacity and health care systems to their limits.^2-7^ An imperative step in the anticipation of delivery of care for a large number of patients would be for hospitals to call upon their surge capacity (ie, capacity to upscale in case of an increased demand for medical resources).^8^ Therefore, as a response to the expected increased demand on hospital care, a national upscale of intensive care unit (ICU) capacity was initiated.^9,10^ In addition, a crisis deliberation was held on March 13, 2020, that aimed to prevent hospitals from being overwhelmed and to guarantee patient safety, as defined by the World Health Organization’s (WHO) statement on organizational leadership.^11^ As a result, patients would be distributed among hospitals and a full transparency would be adhered to regarding hospital’s admission capacity.

Therefore, an existing framework involved in organizing acute care was identified to coordinate the patient distribution. An acute care network, Regionaal Overleg Acute Zorg (ROAZ), formed in 11 regions in the Netherlands, was used to appoint dedicated regional task forces.^12^ In ROAZ region Noord-Holland Flevoland, coordination was handled by physicians trained and experienced in managing mass casualty incidents (MCIs), who took responsibility of the regional distribution of COVID-19 patients in our region during the pandemic. Additionally, a national task force, Landelijk Coordinatiecentrum Patiënten Spreiding (LCPS), was created in order to coordinate interregional transfers.^13^

The importance of equal distribution of resources among multiple institutions, in case of a pandemic, has already been emphasized in the literature.^14,15^ Adhering to a 3-tiered framework in a pandemic has been proposed previously, appointing coordinators on hospital, regional, and national levels.^15,16^ The multilevel collaboration encourages situational awareness among those involved and the possibility to timely decide for patient transfer.^15^ Full transparency among all levels ought to be of great importance.^17^

This review describes the experience of a regional ROAZ network in coordinating the distribution of COVID-19 ICU and clinical patients during the COVID-19 pandemic in the Netherlands, region Amsterdam. By sharing our experience, we aim to emphasize the importance of transparency among hospital, regional, and national coordinators in case of a crisis such as a pandemic. Additionally, the process of creating national, regional, and individual hospital level task forces in a setting of national upscaling is highlighted.

## Methods

### Acute Care Regions

**Figure 1.**
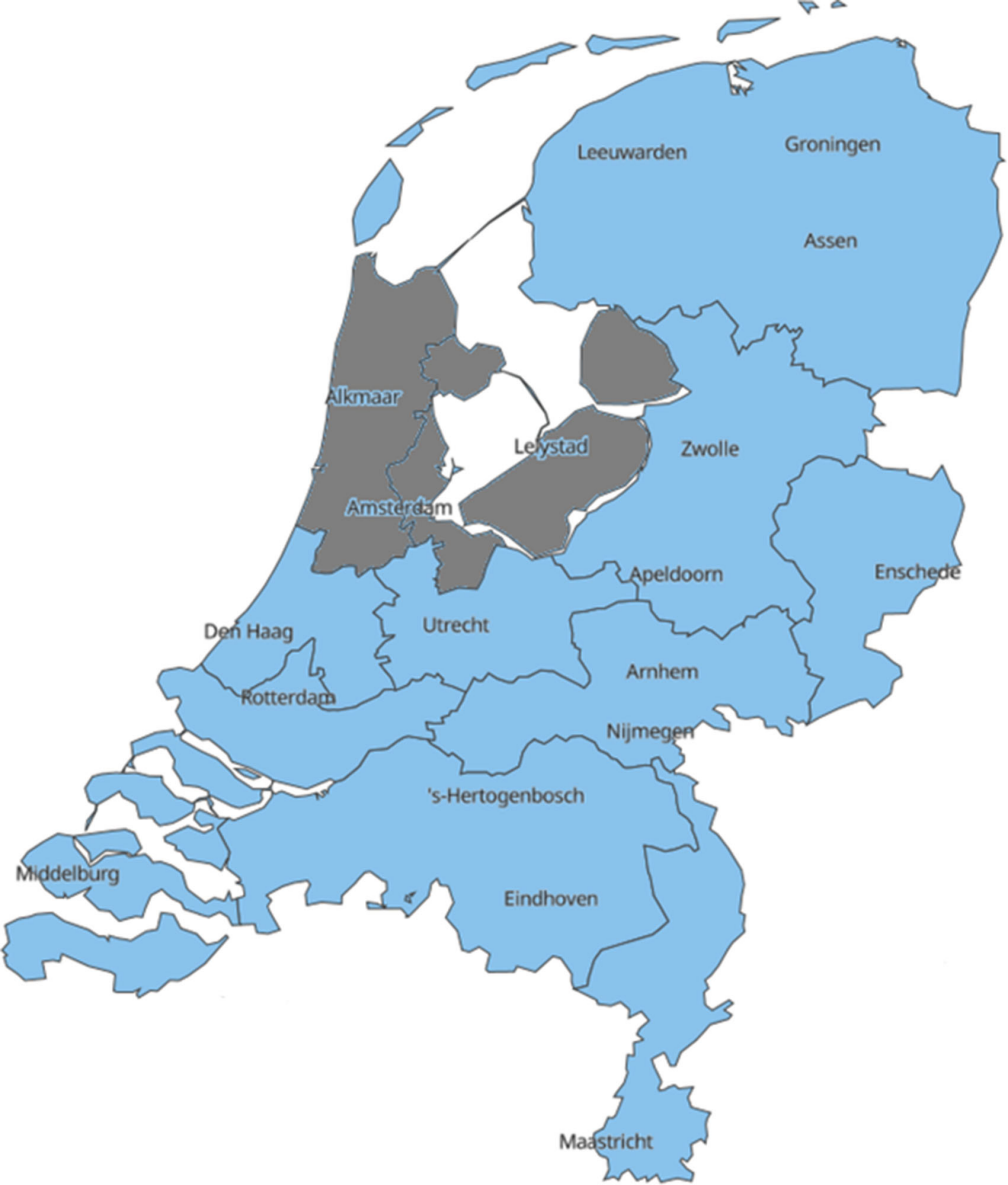
Eleven ROAZ regions in the Netherlands; ROAZ region Noord-Holland, Flevoland is highlighted in gray.^20^

As shown in Figure 1, the medical crisis coordination in the Netherlands is arranged in 11 ROAZ regions, overarched by the body of National Network of Acute Care (Landelijk Netwerk Acute Zorg; LNAZ).^12,18^ Each ROAZ region consists of at least 1 academic level 1 trauma center, regional hospitals, chairmen of the hospital’s board of directors, emergency medical services (EMS), EMS dispatch centers, general practice centers, obstetricians, mental health care facilities, public health service, and Geneeskundige Hulpverleningsorganisatie in de Regio (GHOR).^12^ The collaboration among the involved stakeholders in the integrated acute care chain is characterized by short lines of communication and the ability to consult the acute care portal, a web application showing real-time medical capacity in the region.^19^ The aim of ROAZ is to minimize avoidable delay for acute vitally compromised patients. Their main tasks consist of gaining insights in the providers of acute care in the region, aligning the activities among different acute care providers, and preparing for medical aid in case of disasters and crisis situations. In the North-West region of the Netherlands, a population of about 3.5 million inhabitants is covered by ROAZ region Noord-Holland Flevoland.

### Task Force Setup

In response to the expected saturation of hospital capacity in the region, initially affected by an overflow of COVID-19 patients, the Ministry of Health, Welfare and Sport requested the LNAZ to create a national task force in order to coordinate patient distribution on a national level. Therefore, on March 21, 2020, the National Coordination of Patient Distribution (LCPS) was created.^13^ Similarly, regional task forces for patient distribution on a regional level were created, with each task force represented by a ROAZ region. The coordinating center within the ROAZ region was a large volume academic Level-1-Trauma center. Adding the large hospital bed capacity for COVID-19 patients with expertise in high complexity care, previous experience with crisis coordination and task force organization, Level-1-Trauma centers took responsibility to coordinate the task forces.^21,22^ In our region, ROAZ coordination was performed by physicians with MCI management experience to fulfill the role of 24/7 regional crisis coordinator. Additionally, a team of PhD students and medical students supported with the electronic transfer of patient’s medical records and administrative tasks.

### Coordination Process

**Figure 2.**
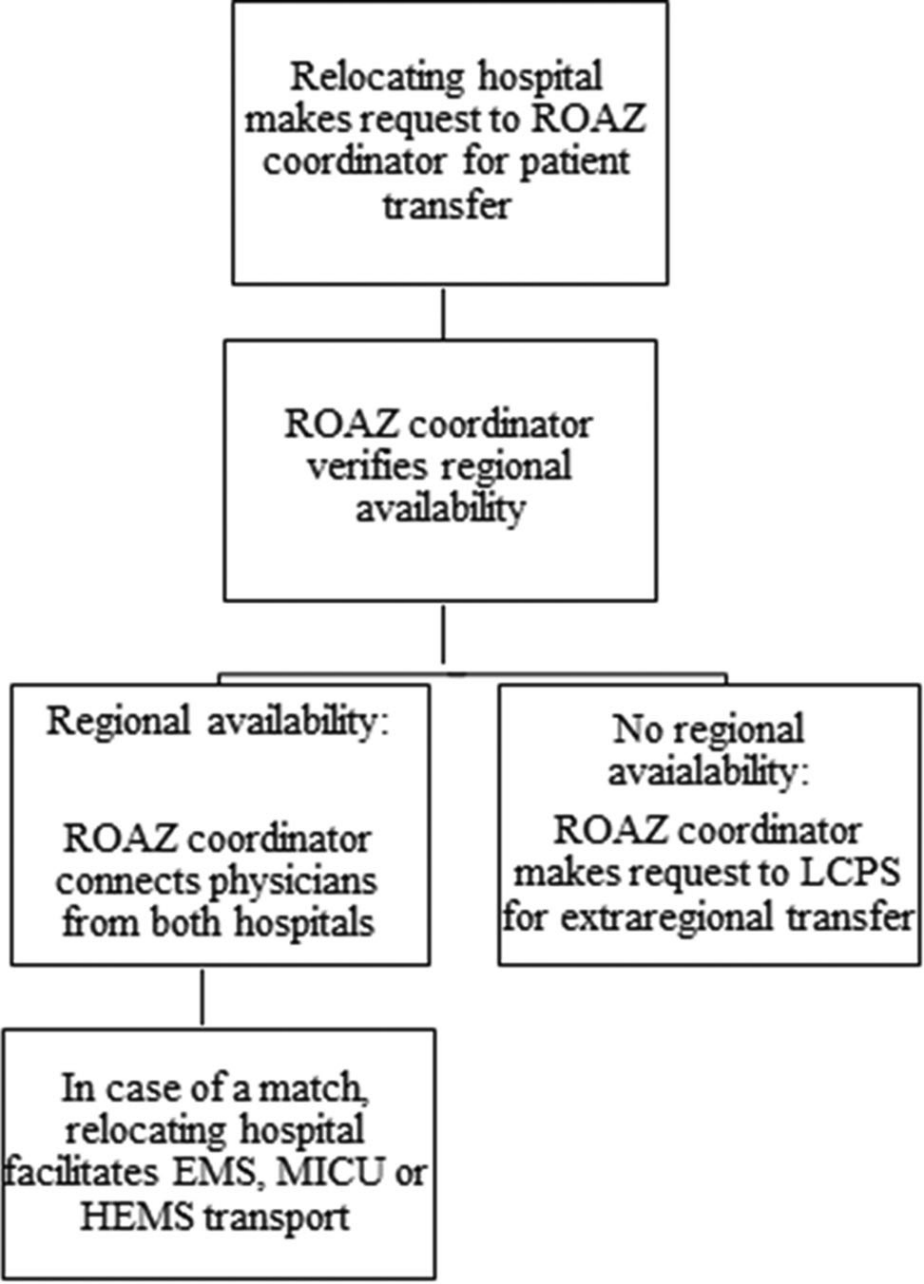
Flow chart of ROAZ activities in case of a patient transfer request.

The ROAZ team endorsed a roadmap to ensure patient safety and minimize errors in judgment. A standardized protocol was developed, guided by the Institute of Medicine’s 6 quality aims (safe, effective, patient centered, timely, efficient, and equitable) for health care (Figure 2).^23^ Three times a day capacity updates were acquired consisting of ICU beds for COVID-19 patients and clinical COVID-19 patient hospital admission availability from hospital crisis coordinators from each hospital within the region. At the same time, to provide insight in the total ICU capacity within the region, daily ICU bed admission capacity reports were gathered for non-COVID-19 patients. Based on this information, the ROAZ coordinator had insight in the current status on regional capacity, upon which transfer requests could be managed. After the capacity update had been received, it was shared with the LCPS, creating full transparency on individual hospital, regional, and national levels.

In case of a transfer request from a hospital within the region, the relocating hospital would send a patient movement request (PMR) form with information regarding necessity of ICU or clinical level care and specifics regarding amount of oxygen provided. The ROAZ coordinator would verify current hospital availability by communicating with other hospitals within the region that had stated available admission capacity in the most recent update (Figure 2). If an admission would be possible, the ROAZ coordinator would connect physicians from both hospitals with one another in order for them to deliberate the medical content and to finalize the transfer. As a result, the treating physicians could focus primarily on patient care.

PMR forms would be sent to both the receiving hospital for medical information and to the LCPS for the sake of a national transfer registration. An encrypted online tool was developed to safely send medical patient information between the sending and receiving hospital and to further lower the administrative burden of the physicians. Transportation would be arranged by the relocating hospital, options including EMS, mobile intensive care unit (MICU), and Helicopter Emergency Medical Services (HEMS), dependent on the extent of care required. In every transfer between hospitals, patient information was registered by ROAZ.

When a patient could not be transferred within the region, a transfer request would be passed on to the LCPS, who would approach ROAZ coordinators from other regions with the request to check their regional capacity. For relocating outside their own region, transportation was arranged by the LCPS. In the case of ICU transportation, the LCPS coordinated the use of MICU and HEMS with the respective ROAZ coordinators. A special arrangement was in place regarding EMS transportations, as EMS from the receiving region would pick up the patient, whereas, during non-pandemic circumstances, a patient would be transported by EMS from the relocating region. Similarly, the LCPS could send a request to ROAZ region Noord-Holland Flevoland to receive a COVID-19 patient from another region. Weekly, 2 to 3 times, national telemeetings were held to discuss the situation in each region among the regional coordinators.

### Data collection

All patient transfers coordinated by ROAZ region Noord-Holland Flevoland between March 21, 2020, and May 22, 2020, were prospectively collected. This acute care region, including Amsterdam, is the largest region in the country with over 3 million inhabitants. Information regarding a patient’s required level of care (eg, ICU or clinical level of care) was obtained. Descriptive data were presented as percentages. In addition, the number of transfers coordinated by the LCPS was retrospectively obtained from the LCPS database. The number of transfers coordinated by our region was compared over time to the number of transfers coordinated by the LCPS and to the total number of COVID-19 hospital admissions in the Netherlands.

## Results

In total, 130 patient transfers were coordinated by ROAZ region Noord-Holland Flevoland. Forty-one (31.5%) transfers originating from outside our region were coordinated to a receiving hospital within our region. Thirteen (10.0%) transfers occurred from inside our region to outside the region, of which 2 transfers had an international destination. Forty-eight (36.9%) transfers took place within our region, of which the majority of patients were transferred to one of the 2 academic trauma centers. In addition, 8 (6.2%) transfers were made to one of our region’s hospitals, but data regarding the relocating hospital were missing. After the demand on hospital capacity decreased, 20 patients were transferred back from ROAZ region Noord-Holland Flevoland to their original relocating hospital (15.4%). In addition, 59 (45.4%) transferred patients required ICU level care and 71 (54.6%) patients required clinical level care.

**Figure 3.**
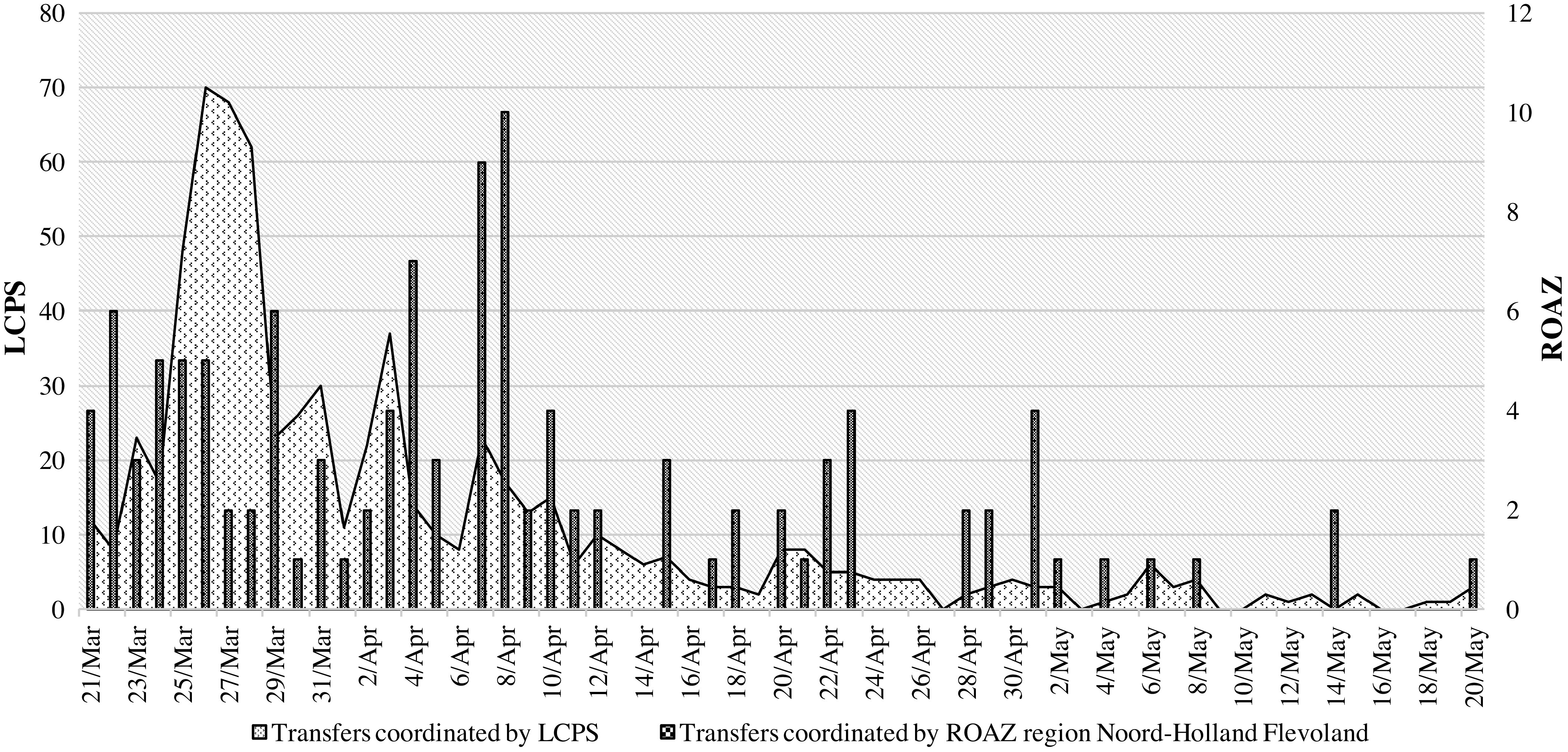
Patient transfers during the COVID-19 pandemic in the Netherlands.

The national task force LCPS coordinated a total of 707 interregional patient transfers in the Netherlands. As illustrated in Figure 3, patient transfers coordinated by ROAZ region Noord-Holland Flevoland and the LCPS both show increases during the months of March and April, with peak incidence on March 26 and April 8.

## Discussion

This review described the coordination process of patient distribution in a setting of nationally upscaled hospital admission capacity in the Amsterdam region of the Netherlands during the COVID-19 pandemic. ROAZ region Noord-Holland Flevoland coordinated a total of 130 patient transfers over a 2-month period, of which 73% were transfers to hospitals in our region. In comparison, the national task force LCPS coordinated a total of 707 interregional patient transfers. Similar increases regarding patient transfers in March and April between our region and the LCPS were found. However, the peak incidence did differ between the coordinating centers. Full transparency was adhered regarding hospital’s admission capacity among crisis coordinators on individual hospital, regional, and national levels. This contributed to the ability to guarantee patient safety for an extraordinary number of patients and prevented hospitals from being disproportionately affected.

From our experience, an imperative aspect in multilevel coordinating is to apply full transparency in hospital admission capacity to the individual hospitals in the region and the national task force.

The importance of transparency within health care systems during a pandemic has recently been emphasized in the literature.^17^ Short lines of communication between facilities and the sharing of information regarding scarce resources (ie, hospital’s admission capacity) are recommended accordingly.^17^ Therefore, within the first days, ROAZ region Noord-Holland Flevoland developed protocols to guide the sharing of capacity, coordination process, and collaboration between hospital, regional, and national levels. The timely setup of protocols has been a contributing factor to the collaboration, resulting in situational awareness and individual task delegation among the involved stakeholders.

During a pandemic, there is no guarantee of the number of patients involved. As all cities are affected and demands on hospital capacity are similar, it is less feasible to acquire the help and resources from other cities or regions. A previous study by Hick et al. emphasized the importance of resource balancing between hospitals and regions.^14^ Coordination between institutions assures a consistent standard of care among regional hospitals. Quick upscaling of capacity should be prioritized, and, during the early days, an appeal should be made on hospital’s surge capacity. Preferably, crisis coordinators and task forces should be appointed, mirroring findings from the study by Sprung et al., who recommended that, during a pandemic, a management system should be created with coordinators at a facility, local, or national level in order to manage resources,^16^ as non-timely transfers of COVID-19 patients and “empty” EMS returns can be considered a waste of valuable resources.

In the Netherlands, although different than MCIs where the primary focus lies within gaining insight in the expected number of casualties and triage, the regional task force coordination was formed by staff experienced with medical crisis coordination.^24^ Therefore, the treating physicians were saved the time of communicating to other hospitals in case of a transfer necessity. A swift collaboration between crisis coordinators from individual hospitals on regional and national levels was feasible due to the already existing network among involved stakeholders.

Few adjustments in workflow design and process were necessary to create a patient safety framework. Although highly complex and timely decisions are common during a pandemic, ethical and human factors cannot be overlooked in the thought process to choose for patient transfer. As a standardized protocol was developed that guaranteed safe and timely patient transfers, complex transfer requests were adequately managed. Factors such as age, mental status, and previous prolonged hospitalization were taken into account by both the treating physician and the ROAZ coordinator in the decision-making process. Physicians from local hospitals could express their PMR to the regional coordinator, who subsequently searched for the nearest hospital available. The treating physician in charge of the medical handover had a final say in whether or not a transfer would be executed.

## Conclusion

Experience with organizing patient distribution on the regional level informs that, in the case of a pandemic, an early upscale of capacity in every hospital in the country is pivotal. This contradicts the management of an MCI, where the early phase is characterized by obtaining knowledge about the number of patients involved. However, as a pandemic affects a country as a whole, upscaling of all capacity avoids hospitals of being affected disproportionately and therefore guarantees patient safety. Furthermore, in the early days of a pandemic, establishing individual hospital, regional, and national crisis coordinators is preferable. Physicians with MCI experience can contribute to combat a pandemic in a coordinating role. Additionally, complete transparency among individual hospital crisis coordinators, regional, and national task forces can facilitate patient distribution and limits the waste of valuable resources by unnecessary long-distance transports.
